# Prevalence and characteristics of Epstein–Barr virus-associated gastric carcinomas in Portugal

**DOI:** 10.1186/s13027-017-0151-8

**Published:** 2017-07-19

**Authors:** Célia Nogueira, Marta Mota, Rui Gradiz, Maria Augusta Cipriano, Francisco Caramelo, Hugo Cruz, Ana Alarcão, Francisco Castro e Sousa, Fernando Oliveira, Fernando Martinho, João Moura Pereira, Paulo Figueiredo, Maximino Leitão

**Affiliations:** 1 Microbiology, Faculty of Medicine of the University of Coimbra, 3004-504 Coimbra, Portugal; 2 CIMAGO, Faculty of Medicine of the University of Coimbra, 3001-301 Coimbra, Portugal; 3 Medical Microbiology, Centre for Neuroscience and Cell Biology of the University of Coimbra, 3004-504 Coimbra, Portugal; 4 Physiopathology, Faculty of Medicine of the University of Coimbra, 3004-504 Coimbra, Portugal; 5 Gastroenterology, University Hospitals of Coimbra, 3000-075 Coimbra, Portugal; 6 Pathological Anatomy, University Hospitals of Coimbra, 3000-075 Coimbra, Portugal; 7 Laboratory of Biostatistics and Medical Informatics, IBILI, Faculty of Medicine of the University of Coimbra, 3000-548 Coimbra, Portugal; 8 Pathology Institute, Faculty of Medicine of the University of Coimbra, 3004-504 Coimbra, Portugal; 9 Department of Surgery, University Hospitals of Coimbra, 3000-075 Coimbra, Portugal; 10 Surgery, Regional Oncology Center of Coimbra, IPOFG, 3000-075 Coimbra, Portugal; 11 Histopathology, Regional Oncology Center of Coimbra, IPOFG, 3000-075 Coimbra, Portugal

**Keywords:** Gastric cancer, Epstein-Barr virus, *Helicobacter pylori*, Clinicopathologic feature, Prognosis

## Abstract

**Background:**

Gastric cancer (GC) is one of the most common malignant tumors of the digestive tract and is the third leading cause of cancer death worldwide. Epstein–Barr virus (EBV) has been associated with approximately 10% of the total cases of gastric carcinomas. No previous study has analyzed the prevalence of EBV infection in gastric cancer of the Portuguese population.

**Methods:**

In the present study, we have analyzed 82 gastric carcinoma cases and 33 healthy individuals (control group) from Coimbra region for the presence of EBV by polymerase chain reaction (PCR) and by in situ hybridization (ISH) for EBV-encoded small RNAs (EBERs). The status of *H. pylori* infection was assessed by serology and by PCR.

**Results:**

EBV was detected by PCR in 90.2% of stomach cancer cases, whereas EBERs were detected in 11%. In our series, EBV-associated gastric carcinoma (EBVaGC) were significantly associated with gender and the majority of them presented lymph node metastasis. These cases were generally graded in more advanced pTNM stages and, non-surprisingly, showed worse survival. *H. pylori* infection was detected in 62.2% of the gastric cancers and 64.7% of these patients were CagA+. On the other hand, the *H. pylori* prevalence was higher in the EBV-negative gastric carcinomas (64.4%) than in those carcinoma cases with EBV+ (44.4%).

**Conclusions:**

The present study shows that prevalence of EBVaGC among Portuguese population is in accordance with the worldwide prevalence. EBV infection seems to be associated to poorer prognostic and no relation to *H. pylori* infection has been found. Conversely, the presence of *H. pylori* seems to have a favourable impact on patient’s survival. Our results emphasize that geographic variation can contribute with new epidemiological data on the association of EBV with gastric cancer.

## Background

Gastric cancer (GC) is one of the most frequent malignant tumors of the digestive tract and is the fifth most commonly diagnosed cancer and the third leading cause of cancer death worldwide (723,073 deaths, 8.8% of the total) [1]. As so, associations to other comorbidities, such as the Epstein-Barr virus and the *Helicobacter pylori* infection, are extremely important as they may be used either clinically as prognosis factor or in basic research to get a deeper understanding of the underlying mechanisms.

The Epstein-Barr virus (EBV) belongs to the *Herpesviridae* family and approximately 95% of the world’s population is infected with it, being the oral route the principal way of infection [2]. In 1997, the International Agency for Research on Cancer (IARC) has classified EBV as a Group I carcinogen for Burkitt’s lymphoma, nasopharyngeal carcinoma and for Hodgkin’s and non- Hodgkin’s lymphoma [3].

The presence of EBV in a patient with gastric cancer was first reported in a case of lymphoepithelioma type by Burke et al. in 1990 [4]. Subsequently, Shibata and Weiss have identified the presence of EBV in 16% of gastric adenocarcinomas in USA [5].Unlike other EBV-associated malignancies, the EBV-associated gastric carcinoma (EBVaGC) is not endemic in any region yet is quite distributed worldwide. In fact, it is emerging as the most common among EBV-associated malignant neoplasms with more than 90,000 patients being estimated to develop GC in association with EBV annually (10% of total GC) [6–8].

*H. pylori* is the major causative agent of gastritis, peptic ulcer disease, mucosa-associated lymphoid tissue (MALT) lymphoma, and GC [9].The clinical outcome of *H. pylori* infection depends on bacterial virulence factors, host susceptibility, environmental and life-style factors [10]. Several *H. pylori* virulence genes have also been identified and among those *cagA* (cytotoxin-associated gene) is one of the most important gene. Infection with CagA strains is associated to higher risk of developing atrophic gastritis and gastric cancer [11, 12].

Some studies have addressed the question if exists a cooperative effect between EBV and *H. pylori* in GC but, their results are inconsistent and conflicting. The present study aims at determining the frequency of EBV-related gastric carcinoma in the Portuguese population and drawing both epidemiological and clinicopathological features of EBV-associated GC in this geographic area relating to *H. pylori* infection.

## Methods

### Patients and samples

A total of 82 patients with gastric cancer who underwent surgical resection at Coimbra University Hospital (HUC) and Regional Oncology Center of Coimbra, IPOFG, SA and 33 patients with non-cancer diseases (control group) who underwent routine surveillance endoscopy at Gastroenterology department of HUC by nonspecific complaints were enrolled in our study. Serum, tumor tissue and their corresponding adjacent non-cancerous mucosa was collected from each gastric cancer patient. Gastric tissue samples and serum were obtained from each individual of the control group.

This study was approved by Ethics Committee of the respective institutions and informed consent was obtained from all individuals. None of the patients received chemotherapy or radiation therapy before surgery. Patient overall survival times were calculated from the date of diagnosis to either the date of death or the last follow up**,** resulting in a follow-up period ranging from 1 to 55 months (mean, 36 months). Those cases lost to follow-up and those ending in death from any other cause than gastric cancer (2 cases) were considered censored data during the analysis of survival rates.

Clinicopathologic data comprise patient age and gender as well as the anatomical site, histological classification according to the Lauren classification system [13], and pathological tumor stage (TNM stage; T: depth of tumor invasion, N: lymph node metastasis, M: distant metastasis) according to the American Joint Committee on Cancer (AJCC) system [14].

### DNA extraction

DNA from tumor tissue and from non-cancerous mucosa was extracted and purified in MagNA Pure Compact equipment (Roche, Germany) using MagNA Pure Compact Nucleic Acid Isolation Kit I (Roche, Germany), according to manufacturer’s instructions. Prior to extraction on the MagNA Pure Compact, tissues were disrupted in Magna Lyser (Roche, Germany) and treated with ATL buffer (QIAGEN, Spain) and proteinase K (QIAGEN, Spain) for 10 min at 65 °C. DNA concentration (A260) and purity (A260/A280) were determined spectrophotometrically (NanoDrop, Thermo Fisher Scientific). DNA was stored at −80 °C for further use.

### EBV real-time PCR

EBV detection was performed using specific primers described by Drouet et al. (1999) [15] that amplify a segment of the *BamH1W* region.

Real-time PCR reactions were carried out on the SmartCycler instrument (Cepheid, USA) in a final volume of 20 μl, containing 2 μl of extracted DNA, 2 μl of FastStart SYBR Green Master kit (Roche, Germany) and 0.4 mM of each primer. Thermocycling conditions were a preheating step of 10 min at 95 °C followed by 45 cycles of 95 °C for 10 s, 59 °C for 5 s and 72 °C for 8 s. Fluorescence was measured at the end of each extension step. Melting analysis was achieved with continuous monitoring of fluorescence from 65 °C to 95 °C at a temperature transition rate of 0.2 °C. A specimen was considered positive if a single melting peak was measured between 88 °C and 89 °C. To validate the amplification process and exclude carryover contamination, positive and negative controls were included in each PCR run.

### EBER1 in situ hybridization

The presence of EBV in gastric cells was identified by the expression of EBV-encode small RNA-1 (EBER1), the most abundant viral product in latently infected cells. In situ hybridization reactions were carried out in an automated system, the BOND -MAX ™ (Leica Microsystems, Wetzlar, Germany) using the staining protocol “ISH protocol A” with an enzymatic pre-treatment with the Bond Enzyme Pre-treatment Kit, according to manufacturer’s instructions. From paraffin-embedded tissues were cut 3 histological sections with 3 μm thick that were mounted on glass slides coated with 3-(aminopropyl) triethoxysilane (Sigma Diagnostics, St. Louis, USA) and used for hybridization with 3 different probes: the EBER Probe, the RNA Positive Control Probe and the RNA Negative Control Probe (Leica Microsystems, Wetzlar, Germany). A sample was considered EBER-1-positive when appeared a dark brown staining in the nuclei of tumor cells, under light microscopy. In each hybridization a positive control, Hodgkin’s lymphoma EBV positive and a negative control, Hodgkin’s lymphoma EBV negative were included. The cases with EBER1 positive signals were classified as EBVaGC group.

### PCR amplification of *H. pylori* and *cagA* gene

Detection of *H. pylori* in gastric samples was accomplished by amplification of *H. pylori* flagella gene. For the *H. pylori*-positive samples, the presence of the *cagA* gene was assessed. Single type PCRs were performed with specific primers described elsewhere [16].

### *H. pylori* serology

IgG antibodies against *H. pylori* were determined with an enzyme-linked immunosorbent assay (ELISA), using a commercial assay (HELICOBACTER PYLORI ELISA IgG, Vircell, Spain), with a sensitivity of 97% and specificity of 100%; according to the manufacturer’s instructions.

### Statistical analysis

The χ2 test and Fisher’s exact test were used to test associations between categorical variables. Cases within the follow-up period were censored either at the time of death or at the last update of the subject. Survival curves were estimated using the Kaplan-Meier product-limit method, and the differences between the survival curves was tested using the log-rank test. All statistical analyses were performed using the Statistical Package for the Social Sciences, version 19 (SPSS Inc., Chicago, IL, USA), and a *p* value <0.05 was considered as statistically significant.

## Results

### Prevalence of EBV positive cases

We evaluated 82 patients (45 males and 37 females; mean age, 66.6; range, 39 to 88 years) with gastric carcinoma and 33 patients (13 males and 20 females; mean age 57.8; range, 30 to 82 years) with non-cancer diseases. Using PCR analysis, EBV DNA was detected in 90.2% of the patients with gastric carcinoma and in 27.3% of the individuals from the control group. However, in situ hybridization showed EBER expression in malignant cells in only 9 patients with gastric cancer (corresponding to 11%) and in one case of the control group (3%). In one of the nine cases EBVaGC, the EBER expression was also detected in non-malignant gastric mucosa. The EBER expression in malignant cells was either uniformly positive or uniformly negative, suggesting that EBV infection may have occurred before malignant transformation and was transmitted to all daughter cells in the neoplastic clone.

### Association between EBV status and clinicopathological characteristics

The clinicopathological characteristics of EBVaGC and EBV-negative gastric carcinomas (EBVnGC) patients are summarized in Table 1. Gender ratio (male/female) was 8:1 in EBVaGC and 1.03:1 in EBVnGC, revealing a significant (*p* = 0.037) association where male shows predominance in EBV positive GC. Although there were no statistically significant differences, EBVaGC were more frequently associated with older age group, notably up to 100% of cases were older than 60 years old versus 75% of EBVnGC.

**Table 1:** Comparison of clinicopathological features between EBV positive gastric carcinomas and EBV negative gastric carcinomas

	EBV positive gastric carcinomas (*n* = 9)	EBV negative gastric carcinomas (*n* = 73)	*P* value
Gender			
Male	8	37	0.037
Female	1	36	
Age (years)			
Mean (range)	70.1 (60–81)	66.2 (39–88)	
18–39	0	1	0.279^§^
40–59	0	17	
≥ 60	9	55	
Tumor location			
Fundus + Body	2	17	1.0
Antrum	5	54	
Cardia	2	2	
Histological type			
Diffuse	2	18	1.0^§^
Intestinal	5	41	
Mixed	2	14	
Lymph node			
Positive	6	47	0.080
Negative	2	26	
pTNM Stage			
I + II	4	42	0.721
III + IV	4	31	
Survival			
≤ 36 months	6	39	0.191
> 36 months	3	32	
*H. pylori*			
Positive	4	47	0.288
Negative	5	26	

^§^ – *p* obtained by Monte Carlo

**Fig. 1:**
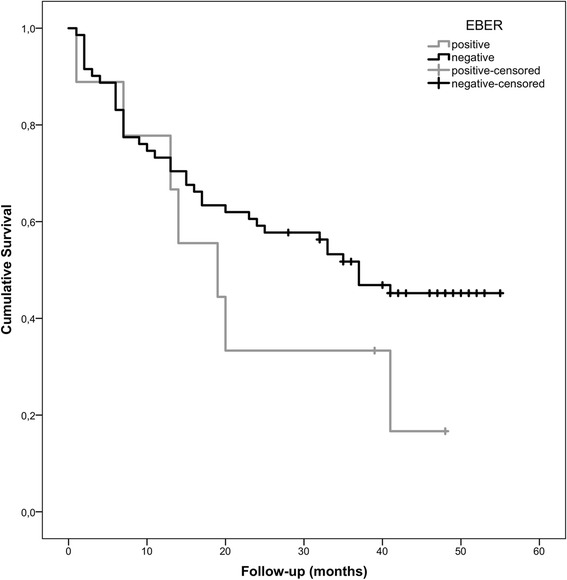
Survival graph of EBV associated gastric cancer and non-EBV associated gastric cancer

Regarding tumor localization, a tendency for the antrum was denoted in both groups, 55.6% in EBVaGC and 74% in EBVnGC; nonetheless, the percentage of tumors at proximal locations was higher in EBVaGC (44.4% vs. 26%). Tumor histology was not related to EBV status since in both groups intestinal type was predominant. Furthermore, the positivity of EBV was not significantly associated with either stage or survival, whilst a slight tendency of EBVaGC having a worse prognosis was noticed. This perception is based on the fact of EBVaGC patients present higher probability of having lymph node metastasis, were typically stratified in more advanced stages and showed poorer survival rates (Fig. 1). Although this trend could be biased by age, gender and the TNM status a deeper analysis reveals that EBV+ subjects tend to present worse survival rate even correcting for that factors. Nonetheless, the attempted statistical models did not present statistical significance for the EBV groups.

### Detection and genotyping of *H. pylori*

The presence of *H. pylori* DNA was identified in 62.2% (*n* = 51) of GC patients and in 54.5% (*n* = 18) of the control group. Comparing the clinicopathological characteristics between the GC *H.pylori* + against the GC *H. pylori*– significant differences due to gender, age, tumor location, histological classification, TMN stage and survival rate were not observed (Table 2). The gene *cagA* was detected in 64.7% (33/51) of the GC cases with *H. pylori* infection and in 38.9% (7/18) at the control group with *H. pylori* infection, which highlights a significant association between the *H. pylori* strains present in tumors to express gene *cagA* (*p* = 0.043). In fact, *H. pylori* strains found in tumors have about 3 times more probability of being CagA+ (OR: IC95% [1.01, 6.66]) than the *H. pylori* strains detected in gastric tissue of healthy individuals.

**Table 2:** Comparison of clinicopathologic variables between *H. pylori*-positive and *H. pylori*-negative gastric cancer patients

	*H. pylori* positive	*H. pylori* negative	*P* value
Gender			
Male	28	17	0.996
Female	23	14	
Age (years)			
Mean (range)	66.0 (44–87)	67.7 (39–88)	
18–39	0	1	0.548^§^
40–59	11	6	
≥ 60	40	24	
Tumor location			
Fundus + Body	13	6	0.561
Antrum	36	23	
Cardia	2	2	
Histologicaltype			
Diffuse	13	7	0.851
Intestinal	29	17	
Mixed	9	7	
pTNM Stage			
I + II	29	17	0.780
III + IV	21	14	
Survival			
≤ 36 months	28	17	0.747
> 36 months	23	12	

^§^ – *p* obtained by Monte Carlo

Seroprevalence of *H. pylori* infection reveals higher rates in GC patients when compared to the control group (85.4% vs 57.6%, *p* = 0.001). The odds ratio of an individual *H. pylori* seropositive to have gastric cancer is about 4 times higher than an individual seronegative (95% CI: [1.71, 10.82]).

Concerning seroprevalence another relationship was observed, namely the association with clinical stage; the majority of *H. pylori* seropositive cases were graded in the less advanced tumors stages (I and II) (*p* = 0.002). The overall survival also follows this trend but with no statistical significance (Log Rank *p* = 0.201, Breslow *p* = 0.141), which is not altered if age, gender and stage status is taken into account.

No significant association was observed between the occurrence of *H. pylori* infection and EBVaGC, since of the nine EBVaGC cases four had *H. pylori* co-infection (*p* = 0.288); moreover the positivity of *H. pylori* in EBVnGC is higher (64.4%) than that found in EBVaGC (44.4%).

## Discussion

Over the past 50 years there has been a decline in gastric cancer (GC) incidence and mortality, however, it still accounts for 6.8% of all malignant tumors [1].

In Portugal, the WHO data for 2012 indicate that the GC is the fifth most frequent malignancy, with 3018 new cases (1834 men and 1184 women), and the third most lethal cancer, as it was responsible for the death of 2285 Portuguese [1].

In the present study, we assessed the status of EBV by PCR and ISH and *H. pylori* infection by PCR and serology in 82 cases of primary gastric carcinomas and in 33 healthy individuals. To the best of our knowledge this is the first report presenting Portuguese casuistry regarding the association of EBV with stomach cancer and, simultaneously correlating the involvement of these two pathogens in gastric carcinogenesis. Using PCR we found that 90.2% of GC were EBV-positive; extremely high positivities of >80% were as well reported in three studies from India [17–19], where EBV detection was also accomplished by PCR; this may be due to the high sensitivity of PCR that amplified indiscriminately EBV DNA present in tumor cells and in tumor-infiltrating lymphocytes. To overcome this issue, it was used EBER in situ hybridization, which is the recommended technique for the detection of EBV in human tissue and tumors because of its high sensitivity and specificity to accurate localize the EBV-infected cells [20]. Hence, by ISH the EBV was detected in 11% of our cases, which is consistent with the prevalence of EBVaGC worldwide, since several studies in the literature report that approximately 10% of gastric carcinomas are associated with EBV [6, 8, 21, 22]. EBER expression was mainly restricted to the tumor tissue, as pointed out by others [23–25], however we found one EBVaGC case with EBER positivity in the non-neoplastic mucosa and one patient of the control group, with chronic pangastritis, was also EBV+ in epithelial cells. This is in line with the observations of others who detected EBV in precancerous lesions [5, 7, 22, 26]. Despite being quite rare, the EBV infection in non-neoplastic gastric mucosa indicates that EBV enters the gastric epithelium at an early stage of gastric carcinogenesis preceding the clonal growth of EBV-infected cells and subsequently the development to carcinoma.

In our study, we found that patients with EBV-positive tumors are predominantly male (8: 1), as corroborated by the majority of published reports [5, 6, 20, 23, 24, 27, 28]. This highest incidence in men can be attributed to both genetic status and lifestyle factors. Earlier studies indicate that eating salty or spicy foods, frequently drinking coffee and high-temperature drinks, exposure to wood dust and/or iron filings and smoking are risk factors for developing EBVaGC [6, 29].

Concerning histological classification and topographic distribution we found a prevalence of intestinal type and tumors located in the antrum, which is consistent with others studies [5, 26, 30] but in disagreement in what regards the characteristic of EBVaGC [20, 24].

Although it has been proposed that the presence of EBV in gastric cancers is associated with a better prognosis [31], former reports are inconsistent. Our results point in the direction that the presence of EBV is a marker of poor prognosis since the majority of our cases have lymph node involvement, are grouped in more advanced stages and, as so, have worse survival.

Taken together, these variations between data might be explained by the contribution of local risk factors, such as geographical and environmental aspects, along with the size and features of the cohort.

It is estimated that *H. pylori* infects half the world population [11, 32, 33] and is responsible for more than 60% of gastric cancer cases [34, 35]. In this series the prevalence of *H. pylori* for both groups, patients with carcinoma and controls, fall within the described in the literature. In the control group *H. pylori* detection rates (DNA - 54.6%, seroprevalence - 57.6%), are close to the average of *H. pylori* infection rate reported worldwide (50%). In GC patients the *H. pylori* DNA was found in 62.2% of the cases, which is also in agreement with its involvement in more than 60% of the total gastric tumors. On the other hand, the serologic positivity was 85.4% which is in line with the value found in another Portuguese study (85.5%) in patients with stomach cancer [36]. These differences in detection rates may be explained by the spontaneous disappearance of the *Helicobacter pylori* during malignant transformation of gastric epithelium, perhaps due to lack of nutrients needed by this bacterium; however, the tumor still occurs after the effective eradication of *H. pylori*; this occurrence has also been described in other studies [37].

In the current study, statistical comparison between the 2 groups revealed that seropositive *H. pylori* status is associated to increasing risk of developing gastric cancer [38, 39] and that *H. pylori cagA*+ strains are more aggressive than *H. pylori cagA*- strains, being also linked to stomach adenocarcinoma progression [11, 12]. Regarding the prognostic value of *H. pylori* status we found a significant association between positive *H. pylori* status and better outcome, since the tumors *H. pylori* + are stratified in early pTNM stages, as observed by others [40–42]. A plausible explanation for this fact is that *H. pylori* may contribute to a more efficient immune response against the tumor by triggering a type-1 T-helper-cell response [43], or it was also suggested that *Helicobacter pylori* antigens mimic the surface molecules of gastric epithelial cells and that would activate a cross-reactivity of autoantibodies against the tumor cells [44]. The involvement of the microsatellite instability is also highlighted, because it has been related with a higher rate of *H. pylori* infection and a better postoperative survival [45].

It has been suggested that EBV and *H. pylori* can be influenced by each other or cooperated together, in a direct or indirect way, in gastric carcinogenesis. In the present study no statistical association was found between EBV infection and *H. pylori* infection once there is no evidence of an *H. pylori* co-participation in the 11% of the GCs that are EBV positive by EBER-ISH. In fact, several studies that address the effect and interaction between them do not detected any association [20, 23, 46–49]. However, there are others publications showing synergism between EBV and *H. pylori* in the pathogenesis of gastric diseases [30, 50–53]. In point of fact, it is suggested two possible mechanisms, first an additional inflammatory response in co-infection and increased tissue damaging by both *H. pylori* and EBV. The studies by Cárdenas-Mondragón et co-workers give evidence of this mechanism; in pediatrics patients they demonstrated that co-infection with EBV and *H. pylori* CagA+ is more associated with severe gastritis than cases with single *H. pylori* CagA+ infection [52], as well as the study with Latin American patients confirm that EBV co-participates with *H. pylori* to induce severe inflammation and increase the risk of progression to intestinal-type GC [53]. The second mechanism pointed out is based on gene products interaction. An in vitro study found that EBV reactivation occurs by the PLC*γ* signalling pathway and *H. pylori* toxin CagA strongly activates PLC*γ* [54]. On the other hand, Saju et al. suggested that host protein SHP1 dephosphorylates CagA, thus preventing its oncogenic activity; however EBV co-infection causes SHP1 methylation and prevents its dephosphorylation activity of CagA and thereby increasing the oncogenic potential of CagA [55].

## Conclusion

We identified 9 cases of EBVaGC (11%) corresponding to the average prevalence of EBVaGC worldwide. EBVaGC was associated with male predominance and seems to emerge as a factor of poor prognosis, while *H. pylori* infection appears to have a protective role in the outcome of GC patients. This results highlight that geographic variation can contribute with new epidemiological data on the association of EBV with GC.

## Abbreviations

AJCC: American Joint Committee on Cancer
*cagA*: cytotoxin-associated gene
EBERs: EBV-encoded small RNAs
EBV: Epstein–Barr virus
EBVaGC: EBV-associated gastric carcinoma
EBVnGC: EBV-negative gastric carcinomas
ELISA: enzyme-linked immunosorbent assay
GC: Gastric cancer
HUC: Coimbra University Hospital
IARC: International Agency for Research on Cancer
ISH: In situ hybridization
MALT: mucosa-associated lymphoid tissue
PCR: polymerase chain reaction
WHO: World Health Organization
